# Evaluation of the Combined Effects of Heat and Lighting on the Level of Attention and Reaction Time: Climate Chamber Experiments in Iran

**DOI:** 10.1155/2018/5171582

**Published:** 2018-05-09

**Authors:** Zohreh Mohebian, Somayeh Farhang Dehghan, Habiballah Dehghan

**Affiliations:** ^1^Department of Occupational Health Engineering, Iranshahr University of Medical Sciences, Iranshahr, Iran; ^2^Department of Occupational Health, School of Public Health, Shahid Beheshti University of Medical Sciences, Tehran, Iran; ^3^Department of Occupational Health Engineering, School of Health, Isfahan University of Medical Sciences, Isfahan, Iran

## Abstract

Heat exposure and unsuitable lighting are two physical hazardous agents in many workplaces for which there are some evidences regarding their mental effects. The purpose of this study was to assess the combined effect of heat exposure and different lighting levels on the attention rate and reaction time in a climatic chamber. This study was conducted on 33 healthy students (17 M/16 F) with a mean (±SD) age of 22.1 ± 2.3 years. The attention and reaction time test were done by continuous performance test and the RT meter, respectively, in different exposure conditions including the dry temperatures (22°C and 37°C) and lighting levels (200, 500, and 1500 lux). Findings demonstrated that increase in heat and lighting level caused a decrease in average attention percentage and correct responses and increase in commission error, omission error, and response time (*P* < 0.05). The average of simple, diagnostic, two-color selective, and two-sound selective reaction times increased after combined exposure to heat and lighting (*P* < 0.05). The results of this study indicated that, in job task which requires using cognitive functions like attention, vigilance, concentration, cautiousness, and reaction time, the work environment must be optimized in terms of heat and lighting level.

## 1. Introduction

Heat exposure is one of the health risk factors for many workplaces especially those located in tropical and subtropical areas such as the southern and southwestern areas of Iran [1, 2]. Heat stress is a situation where the input heat of human body plus the heat produced in the body exceed the heat emitted from the body to the environment [3, 4]. This situation occurs when the worker is exposed to high humidity and temperature, the existence of thermal radiation sources, physical contact with hot objects, and extreme physical activities for a long period of time [5, 6]. Despite the increasing rise of experimental studies in the field of human body's physiologic responses to heat, less attention has been paid to the effects of heat stress on human's cognitive abilities. The recommended levels of exposure to occupational heat stress are trying to basically adjust the limits of exposure to hot environments based on physiological and medical criteria [7, 8]. People's reaction to the increase of their internal body heat is of three kinds: physiological response (e.g., consisting mainly of skin vasodilation and increased production and elimination of sweat), behavioral responses (e.g., decreasing the amount of physical activity, taking off clothes, and avoiding the heat source), and cognitive responses (e.g., decrease in concentration and increase in making errors) [8, 9]. The heat exposure can change individuals' cognitive performance via cognitive exhaustion, lack of comfortable feeling, and reduction in concentration [3, 7, 8, 10].

Lighting level is another physical hazardous agent in workplaces [11] of which recently some evidence regarding nonvisual, biological, and mental effects of light has been detected [12]. However, a few studies have been carried out on the effects of light on attention and performance directly or indirectly [13]. Lighting can be an intense modulator for nonvisual activities like vigilance improvement and cognitive performance of the brain [14]. Moreover, if a person is exposed to inappropriate lighting conditions, the possibility of making mistakes at work would increase [15]. Studies have shown that exposure to higher lighting levels causes an increase in attention, a decrease in sleepiness, and better performance [16]. Adequate lighting will make the employees produce more products with less error, leading to a 10–50% increase in productivity and efficiency of the employees [17]. Therefore, undesirable environmental conditions such as exposure to inappropriate heat and light will influence human error, the occurrence of accidents, and productivity level through the adverse effect on cognitive performance such as attention, concentration, and reaction time [11, 18].

Attention is a set of complicated mental operations, including focus on the goal, care, or endurance and vigilance for a long period of time and changing concentration from one goal to another [19]. Attention can be understood by individuals' number of errors during the test. The higher the person's attention level is during the test, the lower the number of errors will be and vice versa. Attention is also in a close relation to reaction time. In other words, the higher attention level leads to the lower reaction time [20]. Reaction time (RT) is considered as the interval between the perception of the situation and the processing of response in the person [21–23]. Depending on the activity type, attention, and situational awareness, the reaction time in human may last from 0.5 to more than 3 s [24].

Despite the importance of paying attention to this issue, few studies have been carried out on the joint effect of exposure to inappropriate condition of heat and light on cognitive performances; therefore, the present study was aimed to investigate the combined effect of exposure to different levels of heat and lighting on cognitive functions (including attention rate and reaction time).

## 2. Methodology

This empirical study was aimed to investigate the combined effects of heat exposure and lighting levels on the attention rate and reaction time in 2015. All experiments were carried out in a room with controlled atmospheric conditions. Dimensions of the room intended for the experiment were 3 × 4 with a height of 2.8 m, which was equipped with a control system to regulate the operation of heating and cooling system.

### 2.1. Participants

This study was conducted on 33 healthy students of Isfahan University of Medical Sciences (17 M/16 F) with a mean (±SD) age of 22.1 ± 2.3 years. The inclusion criteria for volunteers were being between 19 and 26 years old, having no eye defect, not suffering from color blindness, having no background of consuming of heart disease, diabetes, respiratory disease, and sleep disorders, taking no antidepressants, tranquillizers, antihistamines, anti-Parkinson, or other drugs, having normal hearing, and having no background of cardiovascular disease, breathing problems, and sleep disorders. Informed consent, which was approved by Research Ethics Committee of Isfahan University of Medical Sciences, was obtained from all patients before the experiments. The participants were randomly assigned and selected for doing tests.

### 2.2. Experimental Design

All of the experiments were carried out in a room with controlled atmospheric conditions. The volunteers had to be present 6 times in the chamber and working with the reaction time measuring device. The subjects repeated the test 20 times in 1.5 hours of exposure every 30 minutes. Before the start of the experiment, participants were given the necessary training about performing test. The temperature level of the climatic chamber was set to 22 and 37°C and the lighting levels were 200, 500, and 1500 lux which are supplied by fluorescent tubes (with a color temperature of 4500°C) in the surface of the work desk. Relative humidity was controlled at 20 ± 5% and air velocity was 0.1 m/s in the climatic chamber. Volunteers were exposed to the mentioned levels of heat and light on different days. The sequence of experimental run was randomly selected for each person. They wore the cloth of 0.8 clo during the tests. The wet bulb globe temperature (WBGT) as a reliable index to investigate heat stress ranged from 18.6 to 30°C for these conditions.

### 2.3. Cognitive Tasks

The continuous performance test (Conners CPT; Ravan Tajhiz Sina Co., Iran) and the RT meter (PM-RT16881, Pars Madar, Iran) were used to measure the attention level and reaction time of participants, respectively.

#### 2.3.1. Continuous Performance Test

Continuous performance test is a task-oriented computerized assessment of attention-related problems. The participants performed the test 6 times (each test lasted almost 90 minutes). The main purpose of this test was to evaluate attention or vigilance and impulsivity. In this test, a total of 150 stimuli were presented, 20% of which were the target stimuli (the stimuli to which the subject must respond) and the remaining 80% were the nontarget stimuli. The presentation time of every stimulus was 200 thousandths of a second and the interval between two stimuli was 1 s. The duration of the test was 200 s in total. Omission and commission errors were scored in this test. Omission error occurs when the subject does not respond to the target stimulus and this error is a sign of a subject problem in recognizing the stimulus. This kind of error is defined as a problem in keeping attention and shows the oversight to the stimuli. Commission error occurs when the subject responds to the nontarget stimulus and this error is a sign of failure in preventing the impulses. This kind of error is defined as a problem in emotionalism or impulse or impulsivity control. These two types of errors were counted by computer and with the number of correct responses, the subject's RT to the stimulus was calculated. Thereafter, attention rate was determined by measuring the RT and calculating the number of the individual's errors during the test. By subtracting the correct responses from the total of responses and from the result, a percentage of attention was derived. Reliability or retest coefficients of different parts of the test were in a range of 0.59 to 0.93 [25].

#### 2.3.2. Reaction Time

Reaction time was the other cognitive variable that was measured by Donders' device. The subjects repeated the test 20 times every 30 min in a 1.5 h of exposure. This device measures the RT in simple, selective, and diagnostic situations at one thousandth of a second.

Three types of RT can be realized by this device:

- simple RT, in which a single stimulus is responded by a single reaction;
- selective RT, in which two optical marks with different green, blue, and red colors (two-color selective RT) and two modulation sound stimuli (two-sound selective RT) are used;
- diagnostic RT, when the subject responds to only one stimulus and does not respond to other stimuli which are different from or similar to that stimulus.

The purpose of simple RT test is to determine the minimum reaction time, when the stimulus is unique, simple, and unambiguous, and the response is formed from a simple, ordinary, and automatic movement. The subject sits in front of the indicator lamp, so that she/he cannot see the screen of the reaction time measurement device and movement of the tester in triggering stimulus. Diagnostic and selective RT tests examine the time it takes for the subject to choose the response. At the simple reaction time, the subject's movement was almost automatic. Here, even after a learning that takes longer than simple reaction time learning, the reaction time becomes longer. When the device and the subject were ready, first the subject became familiar on how to respond through some preliminary drills and then the stimulus was presented after the alert and the subject was asked to respond to the stimulus as soon as possible by pushing the manual chassis. In this situation, the timer's counter showed the RT of the subject and the tester recorded it. Finally, the results were charted in a separate table for each subject and the average RT of each person in every step was found. This test has wide application to psychological studies and is used in different studies [25, 26].

### 2.4. Statistical Analysis

Considering the sample size, the researchers would be able to find the variance of 0.07 of standard deviation of attention level in a significant level of 5% with the probability of 80% in two different levels of heat and lighting. The collected data were analyzed using SPSS software, version 16. Student's *t*-tests and one-way and two-way analysis of variance (ANOVA) are used to determine any statistically significant differences of attention rate and RT between the studied environmental conditions.

## 3. Results

Results of continuous performance test for different exposure condition to heat and lighting are presented in Table 1. Mean differences of all performance parameters between different lighting levels are statistically significant for both temperatures of 22°C and 37°C (*P* < 0.05).

Student's *t*-test result also indicated that there is significant difference for all performance parameters in case of temperature at different level of illuminance (*P* < 0.05).

**Figure 1:**
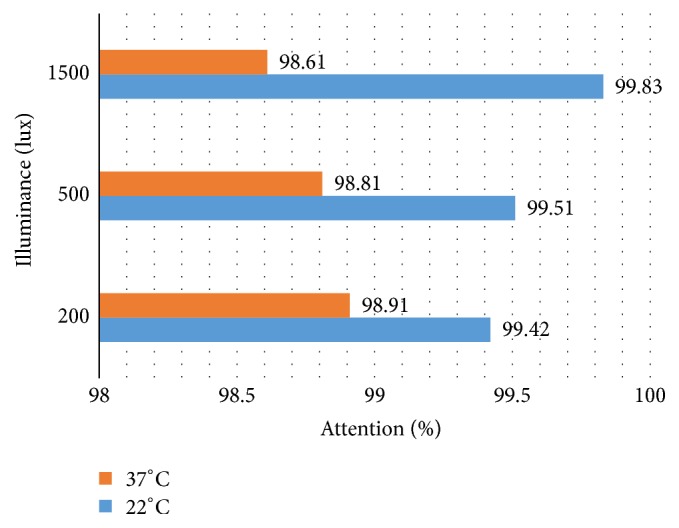
Clustered bar chart of mean attention percent in combined exposure to heat and lighting.

The highest attention rate was obtained in exposure to a 1500 lux of intensity (99.8%) in 22°C temperature. The attention rate had a significant increasing trend in exposure to 22°C temperature with increase in lighting level (*P* = 0.004) (Figure 1). In 37°C, the highest value of attention (98.9%) was observed for illuminance 200 lux and a decreasing trend for attention percent with increase in lighting level was found in 37°C (*P* = 0.002) (Figure 1).

After 1.5 h of simultaneous exposure to heat and light, there was a significant change in response time, correct response, commission error, and omission error (*P* < 0.05) (Table 1). The average number of correct responses was increased in 22°C temperature with increase in lighting intensity. In addition, the average response time, commission error, and omission error had a decreasing trend in 22°C temperature with increase in lighting intensity (*P* < 0.05). In 37°C, for higher level of illuminance, the lower number of correct responses, higher number of commission errors and omission errors, and response time were observed. In certain value of illuminance, performance parameters including commission error, omission error, and response time had the higher values in 37°C than the other one.

**Figure 2:**
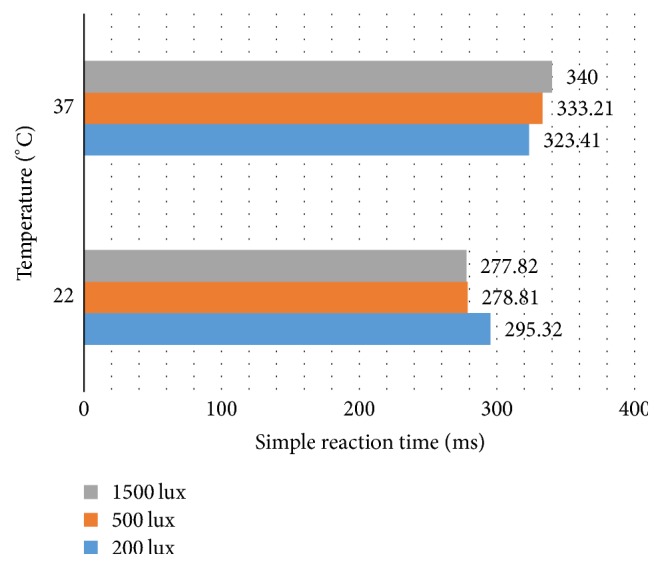
Clustered bar chart of mean simple reaction time in combined exposure to heat and lighting.

Table 2 revealed the mean (SD) of the different parameters of reaction time test in studied experiment conditions. According to Table 2, all types of reaction times in higher temperature have been significantly increased (*P* < 0.05). In 22°C, all types of the reaction time will be decreased with increase in lighting level and in 37°C, the inverse condition is observed. There was the lowest simple reaction time (277.81 ms) and the highest one in combined conditions 22°C and 1500 lux and 37°C and 1500 lux, respectively (Figure 2). The average number of errors increased by increasing the temperature to 37°C. The lowest number of errors (1.66) was in 200 lux and 22°C and the highest number was in 1500 lux and 37°C (Table 2).

Based on two-way ANOVA analysis, the individual and the combined effects of independent variables (temperature and lighting level) on all parameters of performance test were statistically significant (*P* < 0.01), except for the individual effect of lighting level on the number of correct responses and percent of attention (*P* = 0.93) and also the combined effect of temperature and lighting level on the number of correct responses and percent of attention (*P* = 0.205). In terms of reaction time, the individual and the combined effects of temperature and lighting level on all types of reaction time were statistically significant (*P* < 0.001), except for the individual effect of lighting level on two-sound selective RT (*P* = 0.065).

## 4. Discussion

The purpose of this study was to assess the combined effect of heat and lighting on the level of attention and reaction time in climatic chamber. Multiple variables may play a crucial role in decreasing individuals' cognitive performances and increasing human errors. The studies show that when work stress increases in environments with a temperature higher than 24°C and when deep body's temperature increases to more than 38°C, the number of unsafe behaviors and industrial accidents increases [27]. Reaction time is a good indicator to evaluate the effect of heat stress on cognitive performance [7].

In the present study, it was observed that after exposure to different temperature levels [22 and 37°C] in climatic chamber conditions, some changes were noticed in continuous attention, RT, and RT error of the participants. In other words, the decrease in attention and the increase in RT after exposure to heat during the cognitive tests may reflect the effect of the increase in the heat tension of the environment on continuous attention and RT. This finding corresponds to that which found that heat causes an increase in RT [22]. It is also in line with the results of the study conducted by Patterson et al. (1997), who found that RT will be different in exposure to heat, depending on easiness or complexity of the job, and when the temperature increases from 21 to 37°C, RT also increases [28].

Based on the findings of Færevik (2010) [29], human error increases in a warm environmental condition and if someone is asked to focus on a tiring job in such a condition, performance will decrease with the passing of time [30]. Qian et al. (2015) [31] indicated that the bloodstream in the brainstem increases and causes RT to increase, and a decrease of bloodstream in this part in thermal comfort condition causes RT to decrease. Also, based on the reports of McAllen et al. (2006) [32], Fox et al. (2005) [33], Lim et al. (2010) [34], and Liu et al. (2013) [35], the general activity of brainstem increases when skin temperature changes. Moreover, given that the dorsolateral prefrontal cortex acts as the dominant nodes for sobriety and controlling brain performance, decrease in the cerebral blood flow causes a decrease in attention and an increase in errors. Therefore, the decrease in the attention performance may have a relation with the decrease in the bloodstream of the brain in this study. Long-lasting attention is somehow the consumption of cognitive sources and causes mental fatigue and slower reaction and an increase in errors easily [34]. Heat stress makes the participants allocate their attention sources to assessing and coping with heat stressors that cause a decrease in the capacity of processing the information related to their duties that require high attention [36].

In this study, the exposure of individuals to different lighting levels in thermal comfort condition showed that their average attention rate increases as lighting intensity increases. Probably the effect of daze and annoyance of lighting in lighting level of 500 lux has caused an increase in mental fatigue and a slight reduction in attention rate. However, the attention effect may have overcome mental fatigue in 1500 lux of lighting intensity. Therefore, regarding the preparation of stable and equal environmental condition and doing the tests for all individuals, it is necessary to investigate this case in future studies to achieve a final result. Besides, the average RT and error of RT decreased as the lighting intensity increased. This finding corresponds to those of some past studies, including Smolders et al.'s study (2012), who reported that increasing the lighting intensity (1000 lux compared with 200 lux) caused an improvement in the cognitive performance, increase in vigilance, less sleepiness, more energy, shorter RT, and an improvement in continuous attention [37]. According to some previous studies, job exposure to high levels of light can affect some biological parameters (cortisol and melatonin exudation) [38–40]. High level of lighting has been more pleasant to individuals than low level of lighting and being exposed to higher lighting levels at night causes lower exudation of melatonin, increase in physiological motivation, higher mental vigilance, and improvement in continuous attention and cognitive performance. The reason for the improvement in cognitive performance at the same time as the increase in lighting intensity in 22°C temperature in this study may be due to the relationship between lighting, performance, and secretion of melatonin. Melatonin is secreted in poor lighting, causing sleepiness and decrease in mental activities and performance [41]. The present study was conducted during daytime and the time of exposure (1.5 h) to low lighting was at a level that had an inhibitory effect on the exudation of cortisol and caused exudation of melatonin.

In this study, the average simple, diagnostic, two-color selective, and two-sound selective reaction times increased at the same time as the increase in heat and lighting. Furthermore, the attention rate had a decreasing trend simultaneously with the increase in heat and lighting. In other words, exposure to poor lighting intensity and the duration that was allocated in the present study could have an adverse effect on attention; many studies have proven the relationship between lighting, performance, and exudation of melatonin; melatonin exudates in poor lighting, causing sleepiness and decrease in vigilance, mental activities, and functions [41]. According to the results of this study, exposure to 37°C temperature and the increase in lighting intensity caused a decreasing trend in attention rate. Probably, the effect of daze and annoyance of simultaneous increase in lighting and heat has caused an increase in mental fatigue and a decrease in attention rate. This finding corresponds to that of Qian et al. (2015) with the conclusion that when a person is doing cognitive jobs that require high attention, heat stress has an increasing effect on potential mental fatigue [31]. The results of this study are inconsistent with those of some studies conducted previously; [42] reported that increase in heat levels has no effect on attention rate. The reason for this inconsistency is probably attributed to the fact that temperature was increased to 37°C in the present study, which was a higher level compared with the allowable limit, 33°C temperature, for working in a warm environment that was used by Ramsey [42]. Also, the exposure time was 1.5 h in the present study, but it was 1 h in Ramsey's study.

## 5. Conclusion

Attention percent and reaction time are the most basic cognitive responses to external stimuli. The results of the present study demonstrated that heat exposure and the simultaneous increase in lighting intensity cause a decrease in individuals' attention and an increase in their reaction time. On the other hand, our finding confirms the hypothesis that increase in lighting intensity in thermal comfort conditions causes an increase in attention rate and a decrease in reaction time. The findings of this study confirmed the hypothesis of the combined effect of heat exposure and lighting levels on the attention rate and reaction time in laboratory conditions.

The results of this study can provide the information that helps us to improve the thermal conditions and lighting levels in workplaces for job tasks which require using cognitive functions like attention, vigilance, concentration, cautiousness, and reaction time, so that it is necessary that the workplaces have been optimized in terms of thermal conditions and lighting levels.

## Figures and Tables

**Table 1:** Mean (SD) of parameters of continuous performance test in different experiment conditions.

Temperature	Illuminance	Parameter			
		Commission error (number)	Omission error (number)	Response time (ms)	Correct response (number)
22°C	200 lux	0.51 ± 0.71	0.31 ± 0.61	471.11 ± 43	149 ± 0.93
	500 lux	0.35 ± 0.32	0.22 ± 0.31	434.11 ± 22.41	149.1 ± 8.71
	1500 lux	0.31 ± 0.51	0.12 ± 0.33	426 ± 31	149.3 ± 0.61

*P* value		0.001	0.001	0.001	0.032

37°C	200 lux	0.81 ± 0.81	0.71 ± 0.83	541.55 ± 52.11	148.1 ± 1.11
	500 lux	0.91 ± 0.82	0.71 ± 0.83	542.22 ± 55.41	148.2 ± 1.22
	1500 lux	1 ± 0.91	1 ± 1	543.51 ± 82	148 ± 51.51

*P* value		0.002	0.001	0.009	0.002

**Table 2:** Mean (SD) of parameters of reaction time test in different experiment conditions.

Illuminance	Temperature	Parameter			
		Two-color selective (ms)	Two-sound selective (ms)	Diagnostic (ms)	Error (number)
200 lux	22°C	469.21 ± 108.82	469.71 ± 115.73	447.66 ± 117.21	4.7 ± 3
	37°C	491.22 ± 103.21	504.81 ± 122.44	468.34 ± 110	5.91 ± 2.31

*P* value		0.001	0.001	0.001	0.001

500 lux	22°C	455.88 ± 98.42	454.12 ± 107	415.57 ± 97	3.52 ± 2.11
	37°C	496.53 ± 109	505.75 ± 132.44	470.41 ± 114.11	6.11 ± 2.32

*P* value					*P* < 0.05

1500 lux	22°C	436.92 ± 89.21	434.36 ± 106	403 ± 93.34	1.66 ± 1.21
	37°C	514.91 ± 127	525.52 ± 136	487.51 ± 121	8.73 ± 3.52

*P* value		0.001	0.001	0.001	0.001
